# ﻿Karyotype of *Hynobiusfucus* Lai et Lue, 2008, a salamander endemic to Taiwan with comments in memory of June-Shian Lai, a pioneer in studies of mountainous salamanders (Urodela, Hynobiidae)

**DOI:** 10.3897/compcytogen.19.158987

**Published:** 2025-08-14

**Authors:** Koji Iizuka, Hiroyuki Koishi, Nikolay A. Poyarkov, Yoshinori Takeuchi, Yu-Ten Ju

**Affiliations:** 1 Ebara 6th Jr. High School, Koyama 5-20-19, Shinagawa-ku, Tokyo 1420062, Japan Ebara 6th Jr. High School Tokyo Japan; 2 Department of Vertebrate Zoology, Biological faculty, Lomonosov Moscow State University, Leninskiye Gory, Moscow, GSP-1, 119991, Russia Lomonosov Moscow State University Moscow Russia; 3 Bohkai Junior High School, 1-1-33 Nishi-Akashi, Akashi, Hyogo 673-0041, Japan Bohkai Junior Ｈigh School Akashi Japan; 4 Department of Animal Science and Technology, Genetic Laboratory, National Taiwan University, No. 50, Ln. 155, Sec. 3, Keelung Rd., Da’asn Dist.,Taipei 106, Taiwan National Taiwan University Taipei Taiwan

**Keywords:** 58 stream-type, 56 pond-type, karyotype evolution, mainland China, Taiwanese salamander

## Abstract

The chromosome number of *Hynobiusfucus* was found by Lai and Lue (2008) to be 2n = 58, displaying a karyomorph similar to those previously reported in stream-type salamanders from Taiwan. Based not only on cytogenetic features but also on developmental characteristics such as the embryonic stage and the presence of interdigital membranes during limb formation this species can be confidently classified as a lotic stream-type salamander. Morescalchi (1975) proposed that karyotype evolution in families of urodeles tends to proceed from higher to lower chromosome numbers. Our findings from Taiwan suggest karyotype evolution within the genus *Hynobius*, that is, the chromosome number of this species may have increased from 2n = 56 in the pond-type ancestor to 2n = 58 in this stream-type lineage.

## ﻿Introduction

**Figure 1.**
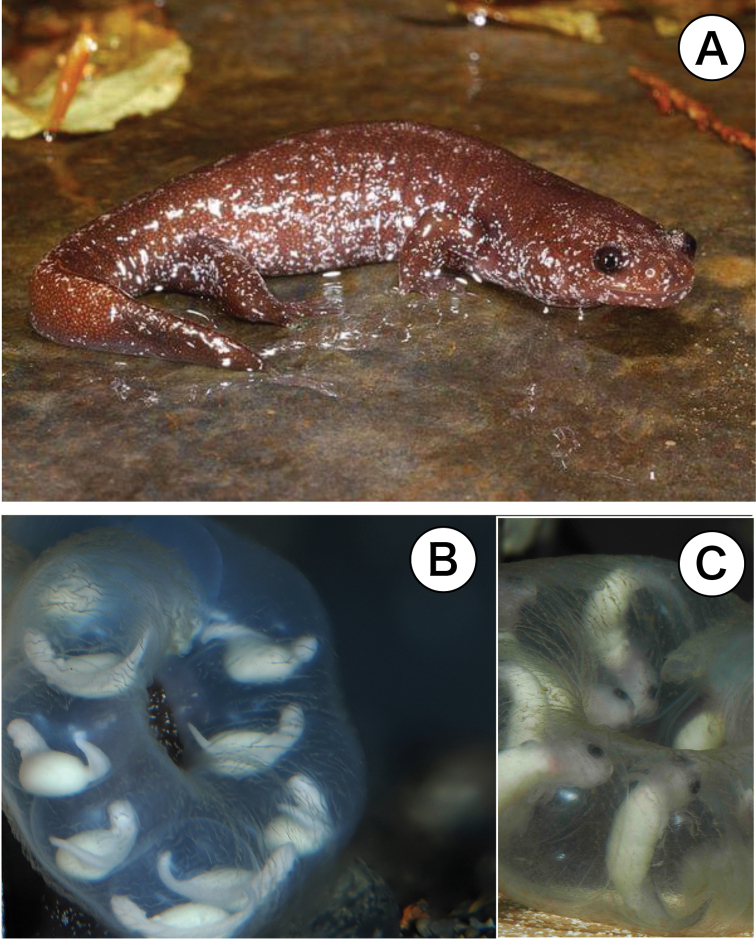
**A***Hynobiusfucus* photographed by Dr. June-Shian Lai who described a new species from Taiwan mountains in 2008. The hindlimbs have four toes. The morphological changes of *H.fucus*, male adult during the breeding season shows lentic characteristics such as a flattened tail fin. **B** Tail-bud stage embryos that were laid on January 1^st^, 2025. *H.fucus* is undoubtedly a lotic stream-type salamander. **C** Final stage of embryos with visible black eyes just prior to hatching. After this, the forelimbs form.

**Figure 2.**
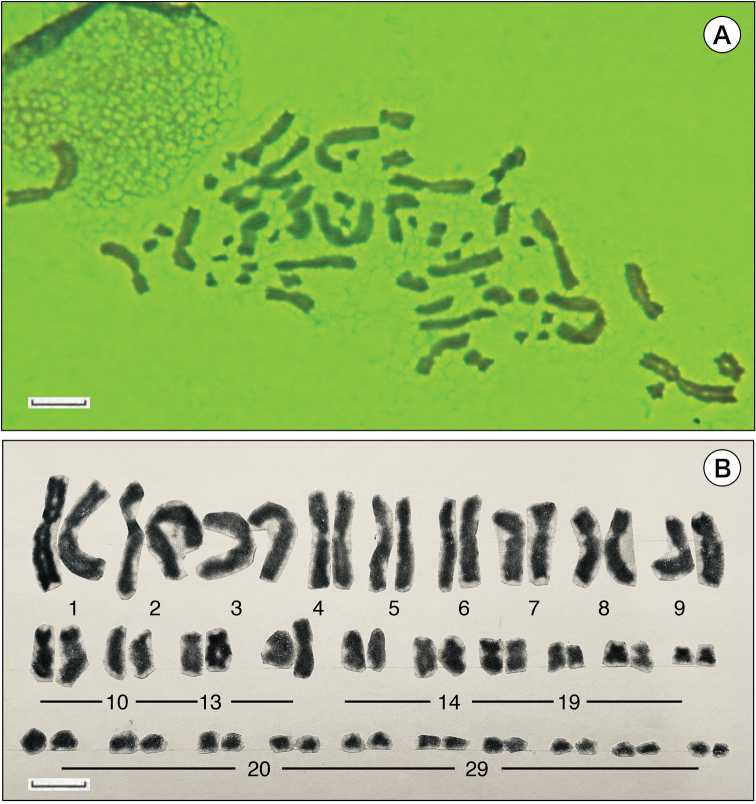
**A** Chromosome spreads prepared from intestinal epithelium of *H.fucus* immature juvenile, unknown sex. Diploid chromosome numbers are 2n = 58. This chromosome number is characteristic of many stream-type species of the genus *Hynobius*. **B** Karyotype with Giemsa-stained chromosomes. Chromosome constitutions are as follows, macro: 13 bi-armed 0 uni-armed; micro: 6 bi-armed 10 uni-armed. Scale bars: 10 μm (**A, B**).

This study is in memory of the late Dr. June-Shian Lai of National Taiwan Normal University, who provided us with research materials (Iizuka 2016) and was a pioneer in salamander research in Taiwan (Lai and Lue 2008). There have been three reports on the chromosomes of Taiwanese salamanders: *Hynobiusarisanensis* Maki, 1922 (Seto and Utsunomiya 1987), *Hynobiusformosanus* Maki, 1922 and *Hynobiussonani* (Maki, 1922) (Iizuka et al. 1989), and chromosome banding of the above species (Iizuka and Kakegawa 1989). Subsequently, Lai and Lue (2008) described two new species from northern Taiwan, namely *H.fucus* Lai et Lue, 2008 (Fig. 1A) and *Hynobiusglacialis* Lai et Lue, 2008, but no reports on the chromosomes of these new species have been published. Here, we report chromosomal information obtained from chromosomal sampling of *Hynobiusfucus* Lai et Lue, 2008 (Fig. 2A, stored in the database) prepared in July 2009. Fortunately, we obtained embryonic evidence based on laboratory breeding in the winter season, i.e., tail-bud stage embryos of *H.fucus* that were laid on January 1^st^, 2025. (Fig. 1B). We also present the karyotypes of the three stream-type Taiwanese species reported to date as well as those of the genus *Hynobius* inhabiting various parts of East Asia, especially mainland China across from Taiwan, and compare them with the results of this study, including the previous Taiwanese salamanders. In addition, building on the recently reported nuclear DNA analysis results of the five Taiwanese species (Chen et al. 2024), we will discuss the karyotype evolution of conclude *H.fucus* and the other Taiwanese species and the genus *Hynobius* five species from mainland China, including developmental physiology.

## ﻿Material and methods

The immature *H.fucus* specimen used for the observations was collected in July 2009 by Chan Yi-Hao and Nikolay Poyarkov under the supervision of the late Dr. June-Shian Lai from the foot of the Hsueh-Shan Mountains in the Guanwu, Miaoli county in Taiwan at an altitude of about 1,300 meters. The specimen was selected because it had a good somatic metaphase figure when stained with Giemsa. We prepared metaphase chromosomes from gastrointestinal epithelial cells using the method of Kezer and Sessions (1979). The animals were injected with 0.1 ml of 2 mg/ml colchicine solution per 1.0 g body weight and dissected 48 hours later. Cells taken from the digestive epithelium were treated with distilled water, fixed with Carnoy’s solution (1 part acetic acid, 3 parts methanol), and separated with 45% acetic acid solution. Slides were prepared by air-drying and staining with 4% Giemsa solution, then observed and photographed under a microscope. Chromosome division images were photographed with an Olympus Camedia C-2000 digital camera equipped with a 2.11 million pixel CCD (total number of pixels) mounted on an Olympus BH2 (BHS) microscope equipped with a Ryoko-sha attachment lens.

## ﻿Results

*H.fucus* is undoubtedly a lotic stream-type salamander, sharing features with rheophilic stream-type salamanders from Japan and Taiwan, like large egg size, nutritious egg yolks, and a cream-colored body, which is unprotected against ultraviolet light due to a lack of melanin (Fig. 1B, C, Kakegawa et al. 1989; Vassilieva et al. 2015). A sufficient number of metaphase cells were obtained (Fig. 2A) and, since an adequate number of chromosome division images were found thanks to Giemsa staining, karyotypes were analyzed (Fig. 2B, Table 1). The karyotype analyzed from juvenile somatic cells of unknown sex consisted of 29 pairs of homologous chromosomes. The chromosome number was 2n = 58, like the other three rheophilic stream-type Taiwanese species (Fig. 2B, Table 2, Seto and Utsunomiya 1987; Iizuka et al. 1989). The 29 pairs of homologous chromosomes consist of 9 pairs of large bi-armed chromosomes, 4 pairs of medium bi-armed chromosomes, 6 pairs of small bi-armed chromosomes, and 10 pairs of small uni-armed chromosomes (Fig. 2B, Iizuka and Kakegawa 1989), resulting in a fundamental number (FN: number of chromosome arms) of 96.

**Table 1.** Quantitative characteristics of chromosomes of *Hynobiusfucus*.

Chromosome number	Relative length	Arm ratio	Centromere position
1	9.63	1.28	m
2	9.30	1.37	m
3	8.62	1.33	m
4	8.29	1.28	m
5	8.00	2.13	sm
6	6.90	2.17	sm
7	6.19	1.84	m
8	6.13	2.0	sm
9	5.78	2.4	sm
10	4.01	2.79	sm
11	3.34	2.03	sm
12	2.51	2.95	st
13	2.31	1.72	m
14	2.27	1.94	sm
15	2.28	2.15	sm
16	2.01	1.51	m
17	1.32	1.07	m
18	1.18	1.17	m
19	0.85	1.43	m
20	1.70	–	t
21	1.21	–	t
22	1.10	–	t
23	0.94	–	t
24	0.79	–	t
25	0.74	–	t
26	0.74	–	t
27	0.71	–	t
28	0.62	–	t
29	0.54	–	t

**Table 2.** Cytological data in hynobiid salamanders from Taiwan and mainland China. FN: Fundamental number. S: Stream-type P: Pond-type.

Species		Collecting locality	Chromosome numbers	FN	Breeding habitat	Biarmed macro micro		Uniarmed macro micro		Reference
Taiwan	*Hynobiusfucus*	Guanwu, Miaoli	58	96	S	13	6	0	10	Pesent study
	*H.arisanensis*	Alishan, Chiayi	58	96	S	13	6	0	10	Seto and Utsunomiya 1987; Iizuka and Kakegawa1989
	*H.sonani*	Songyuan, Nantou	58	96	S	13	6	0	10	Iizuka et al. 1989; Iizuka and Kakegawa 1989
	*H.formosanus*	Tsuifeng, Nantou	58	94	S	13	5	0	11	Iizuka et al. 1989; Iizuka and Kakegawa1989
China	*H.bambusicolus*	Wuli, Fujian	56	94	P	13	6	0	9	Zeng et al. 1997
	*H.amjiensis*	Anji, Zhejiang	56	92	P	13	5	0	10	Zeng et al. 1997; Ikebe et al. 1998
	*H.yiwuensis*	Zhenhai, Zhejiang	56	92	P	13	5	0	10	Ikebe et al. 1998
	*H.guabangshanensis*	Guabangshan, Henan	56	92	P	13	5	0	10	Xiong et al. 2008
	*H.maoershanensis*	Maoershan, Guangxi	56	92	P	13	5	0	10	Qing et al. 2008

## ﻿Discussion

**Figure 3.**
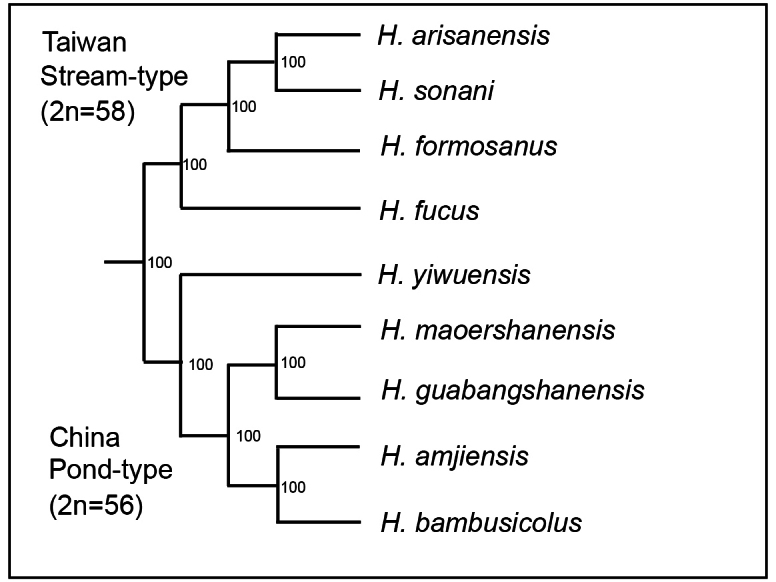
A phylogenetic tree showing the phylogenetic relationships of the four Taiwanese and five Chinese salamander species shown in Table 2, created based on the DNA data of Wang et al. (2023) and Chen et al. (2024).

*H.fucus* lives in mountainous areas at altitudes of 1300–2290m (Chen et al. 2024), and the characteristics of its eggs indicate that it is a rheophilic stream-type salamander. As shown in the photo, the morphological characteristics of the developing embryo are typical of stream-type salamanders, but during the breeding season, the tail fin of adult males becomes vertically flattened, typical of pond-type salamanders (Fig. 1A, Vassilieva et al. 2015; Chen et al. 2022). In addition, DNA analysis of the five species of salamanders in Taiwan confirmed that all five species are monophyletic, sharing the same ancestor. In particular, *H.fucus* is located on the outermost branch of the molecular phylogenetic tree and is estimated to be close to the ancestral type because it lives in lowlands (Fig. 3, Chen et al. 2024). Five species of salamanders with 56 chromosomes live in mainland China, which belongs to the Eurasian continent (including the Korean peninsula) (Seto and Iizuka 1993; Qing et al. 2008). To date, no salamanders with 58 chromosomes have been found in mainland China (Table 2, Zeng et al. 1997; Ikebe et al. 1998; Xiong et al. 2008; Qing et al. 2008). A phylogenetic tree showing the phylogenetic relationship between the four Taiwanese and five Chinese salamanders discussed in Table 2 is shown in Fig. 3 (Wang et al. 2023; Chen et al. 2024).

**Figure 4.**
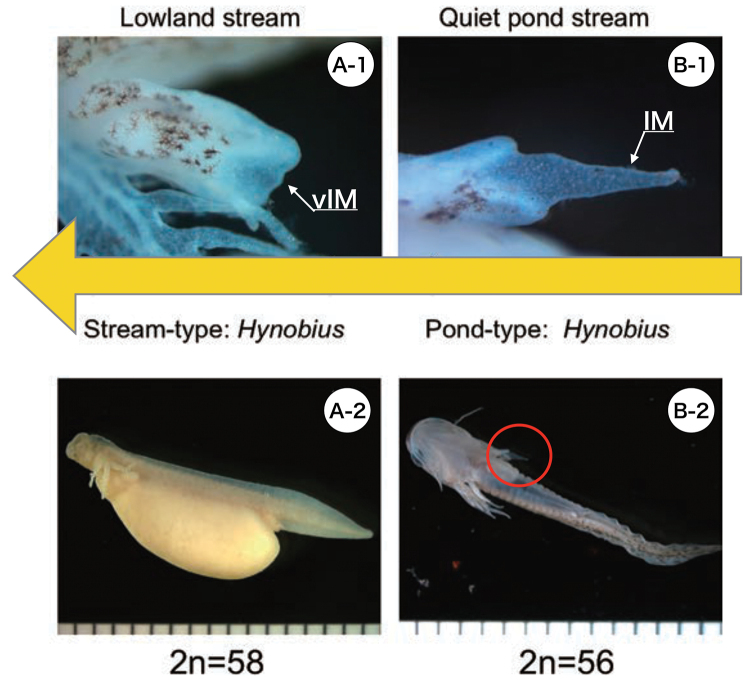
Stream-type **(A)** and pond-type **(B)** salamanders living in Honshu, Japan. **A-1** Enlarged view of the forelimb **A-2** Tailbud embryo, stream-type salamander *Hynobiuskimurae* (2n = 58). The arrow indicates the vIM of the forelimb **B-1** Enlarged view of the forelimb **B-2** Post-hatching larva, pond-type *H.tokyoensis* (2n = 56). Similarly, the arrow indicates the IM. The area circled in red in **B-2** is the IM of the forelimb. The large yellow arrow indicates the direction of evolution.

Therefore, the following is thought to have happened: The ancestors of salamanders invaded from mainland China, eventually living in mountainous areas at altitudes of 1,300 to 3,600 m, and then speciated into five species (Iizuka 2009; Iizuka et al. 2016). The chromosomes of *H.glacialis*, which lives at 3,400 m near the highest peaks, are unknown, but four species, including *H.fucus*, have a chromosome number of 2n = 58 (present study; Iizuka et al. 2016). *H.fucus* (2n = 58) shows a karyotype most similar to that of the newly described pond-type *Hynobiusbambusicolus* Wang et Qiu, 2023 (2n = 56) from Fujian Province, mainland China, except for one pair of small chromosomes (Table 2, Zeng et al. 1997; Ikebe et al. 1998; Xiong et al. 2008; Qing et al. 2008; Wang et al. 2023). What is noteworthy here is the microchromosome. Seto (1994) also noted the number of microchromosomes. *H.bambusicolus* (2n = 56) has six pairs of bi-armed microchromosomes (=BMC) and nine pairs of uni-armed microchromosomes (=UMC). The *H.fucus* (2n = 58) used in this study has six pairs of BMC and ten pairs of UMC (Table 2). Thus, speciation is considered to have occurred in the direction of increasing one pair of UMC. A similar phenomenon is considered for the remaining two species found in Taiwan, *H.arisanensis* and *H.sonani*. Both species have 10 pairs of UMC (Table 2). Thus, speciation is considered to have occurred in the direction of increasing one pair of UMC from the ancestor of *H.bambusicolus* (2n = 56) in the three species found in Taiwan (2n = 58) (Table 2). On the other hand, the four pond-type salamanders found in mainland China (2n = 56) have 5 pairs of BMC and 10 pairs of UMC (Table 2). One species found in Taiwan, *H.formosanus* (2n = 58), has 5 pairs of BMC and 11 pairs of UMC (Table 2). It is also believed that *H.formosanus* differentiated from the ancestral lineage of the four pond-type species in mainland China by increasing the number of UMC pairs by one. It is believed that, for some reason, the five 58 chromosome salamander species were born from the 56 chromosome salamander species by increasing the number of UMC pairs by one.

Chen et al. (2024) stated that all five Taiwanese salamander species are monophyletic with the same ancestor. On the other hand, karyotype analysis, including this study, has shown that the three Taiwanese species, including *H.fucus*, evolved from the ancestral lineage of *H.bambusicolus* in mainland China, and *H.formosanus* in Taiwan evolved from the ancestral lineage of four species, including *Hynobiusamjiensis* Gu, 1992 in mainland China. In other words, it is believed that the five Taiwanese species (2n = 58) were born from two pond-types of ancestral lineages (2n = 56). However, if we go back further, the Chinese mainland species may be narrowed down to being monophyletic. It seems to be possible that the evolution of karyotypes in families of urodeles progressed from those with more chromosomes to those with fewer chromosomes (Morescalchi 1975). Moresecalchi does not discuss changes in chromosome numbers within the genus. This study in Taiwan suggests that the 56 and 58 chromosomes evolved from those with fewer chromosomes to those with more chromosomes in the genus *Hynobius*, that is, from 56 to 58.

Fig. 4 shows the interdigital membrane (IM) of 56 chromosomes *Hynobius* and the vestigial interdigital membrane (vIM) of 58 chromosomes *Hynobius*. The *Hynobius* which lives in ponds and has 56 chromosomes is thought to use its pointed IM to fix its body to the bottom of the pond, allowing it to respond to slight currents and skillfully catch small animals for food (Fig. 4B, Iizuka et al. 2016). On the other hand, the *Hynobius* which lives in streams and has 58 chromosomes is thought to have lost its need for a pointed IM during the early stages of its larval period because it can grow its body inside the egg sac using the yolk, causing the IM to degenerate (Fig. 4A, Iizuka et al. 2016). In 56-chromosome *Hynobius* and 58-chromosome *Hynobius*, IM and vIM disappear as the third toe develops. Since the morphology of IM and vIM of *Hynobius* from Taiwan and China is very similar to that of IM and vIM of *Hynobius* from Japan, the figure shows the Japanese specimens. Based on these facts, it can be inferred that *Hynobius* evolved from 56 chromosomes to 58 chromosomes.

Furthermore, as a future challenge it seems to be necessary to carry out practical experiments to obtain direct scientific evidence (possibility) for the increase in a single pair of uni-armed microchromosomes. To this end, we recommend conducting molecular hybridization experiments on chromosomes to compare the between the 56 chromosome, pond-type *Hynobius* species from Fujian Province (across from Taiwan) and the 58 chromosome, stream-type *H.fucus* using techniques such as chromosome painting and FISH (fluorescence *in situ* hybridization) with chromosome-specific probes.

## ﻿Author contributions

K. Iizuka, H. Koishi, N. Poyarkov, Y. Takeuchi, Y-T. Ju contributed equally to this work.
